# Superior mesenteric artery syndrome following spine surgery in idiopathic adolescent scoliosis: a systematic review

**DOI:** 10.1186/s40001-024-02002-3

**Published:** 2024-08-08

**Authors:** Gaetano Pappalardo, Enrico Pola, Fracesca Alzira Bertini, Luigi Aurelio Nasto, Jörg Eschweiler, Luise Schäfer, Filippo Migliorini

**Affiliations:** 1 Department of Spine Surgery, Oberlinhaus, 14482 Potsdam, Germany; 2 Orthopaedics and Traumatology Division, Multidisciplinary Department of Medical-Surgical and Dental Specialities, University of Campania “Luigi Vanvitelli” School of Medicine, 80138 Naples, Italy; 3 Department of Orthopaedic and Trauma Surgery, Academic Hospital of Bolzano (SABES-ASDAA), 39100 Bolzano, Italy; 4 Department of Orthopaedic, Trauma, and Reconstructive Surgery, BG Hospital Bergmannstrost Halle, Halle, Germany; 5 Martin Luther University Halle-Wittenberg, Halle, Germany; 6 Department of Orthopaedic and Trauma Surgery, Eifelklinik St.Brigida, 52152 Simmerath, Germany; 7 Department of Orthopaedic, Trauma, and Reconstructive Surgery, RWTH University Hospital, Pauwelsstraße 30, 52074 Aachen, Germany; 8 Department of Life Sciences, Health, and Health Professions, Link Campus University, Rome, Italy

**Keywords:** Superior mesenteric artery syndrome, Wilkie syndrome, Cast syndrome, SMAS, Adolescent, Scoliosis

## Abstract

Superior mesenteric artery syndrome (SMAS) is a rare and unpredictable complication after correction spine surgery for adolescent idiopathic scoliosis (AIS). The management of this condition is poorly investigated, with controversial outcomes. This investigation systematically reviewed current evidence on pathogenesis, risk factors, management, and outcomes of SMAS following correction spine surgery for AIS. The present systematic review was conducted according to the 2020 PRISMA statement. All the included investigations reported SMAS presentation following scoliosis correction surgery in AIS. 29 articles with 61 eligible patients were included in this review. The mean age of the patients was 15.8 ± 7.2 years. The mean weight was 45.3 ± 8.0 kg, the mean height 159.6 ± 13.6 cm, and the mean BMI 16.5 ± 2.9 kg/m^2^. The mean duration of the treatment for SMAS was 21.6 ± 10.3 days. The mean interval between spine surgery and symptoms of SAMS was 69 days, with high between-studies variability (3 days to 4 years). Prompt identification of risk factors and an early diagnosis are necessary to manage SMAS and reduce the risk of complications. Additional investigations are required to establish risk factors and diagnostic criteria.

*Level of evidence* Level IV, systematic review.

**Supplementary Information:**

The online version contains supplementary material available at 10.1186/s40001-024-02002-3.

## Introduction

The superior mesenteric artery syndrome (SMAS), also known as “Wilkie syndrome”, is a complication of scoliosis surgery [1–3]. This clinical condition was first illustrated by Carl von Rokitasky in 1861 and later in greater detail by Wilkie in 1927 [4–7]. The reported incidence of SMAS after scoliosis surgery is approximately 0.013–4.7% [8–11]. The aetiology of SMAS is a change of the anatomical relationship between the third part of the duodenum, lumbar spine, and superior mesenteric artery (SMA) [12, 13]. The SMA arises from the anterior aspect of the aorta at the level of L1–L2 vertebral bodies [14, 15]. At its origin, the SMA is encased in fat and lymphatic tissue and descends downwards the mesentery [8, 11, 16]. On the other hand, the duodenum traverses the aorta at the level of the L3 vertebral body and is suspended by the ligament of Treitz between the aorta and the SMA [8, 16, 17]. Any factor which modifies this anatomical relationship could lead to an extrinsic compression of the duodenum [11, 17–19]. SMA syndrome presents clinically unspecific simulating other factors and causes of upper gastrointestinal obstruction [4–7]. The correct diagnosis of SMAS may be challenging, requiring a proper correlation of clinical signs and symptoms with radiographic findings [4–7]. A timely diagnosis and appropriate management of SMAS is recommended since delayed treatment has a mortality rate of 33% [1, 6, 20]. Its management is conservative initially, with the rationale of weight gain to increase the retro-peritoneal fat and the aortomesenteric angle [13, 21, 22]. If the conservative therapy fails, surgical options are represented by gastrojejunostomy, duodenojejunostomy, or Ladd procedure [13, 21, 22].

Evidence on SMAS is limited. A proper therapeutic algorithm and internationally accepted guidelines are missing. This investigation systematically reviewed current evidence on pathogenesis, risk factors, management, and outcomes of SMAS following correction spine surgery for adolescent idiopathic scoliosis (AIS).

## Methods

### Eligibility criteria

All the clinical studies investigating SMAS following spine surgery for AIS were accessed. According to the author´s language capabilities, English, German, Italian, and Spanish articles were eligible. According to the Oxford Centre of Evidence-Based Medicine, we included only studies with levels I to IV of evidence [23]. Studies published in grey literature or without full text were not eligible. Opinions, letters, and editorials were not considered. Only clinical studies were eligible. Missing quantitative data under the outcomes of interests warranted the exclusion of the study.

### Search strategy

This study was conducted according to the Preferred Reporting Items for Systematic Reviews and Meta-Analyses: the 2020 PRISMA statement [24]. The following framework was used to guide the literature search:

- P (Problem): SMAS following AIS;
- I (Intervention): scoliosis correction surgery;
- C (Comparison): height, weight, length of fusion as risk factor for SMAS;
- T (Time): interval between the surgical treatment and presenting the symptoms for SMAS.

In December 2023, the following databases were accessed: PubMed, Web of Science, and Google Scholar. No time constraint was set for the search. The medical subject headings (MeSH) used in each database for the search are reported in the appendix. Additional filters were not used for the database search.

### Selection and data collection

Two authors (G.P. and L.S.) independently executed the database search. All matched titles were screened by hand, and the abstract was examined if suitable. The full text of the abstracts matched the topic and was accessed. Studies without accessible or available full text were not included. A cross-reference of the bibliography of the full-text manuscripts was also performed for the inclusion. Authors debated controversies. A third senior author (F.M.) took the final decision in case of additional differences of opinion.

### Data items

Two authors (G.P. and L.S.) separately performed data examination and analysis. The following data at baseline were extracted: author, year and journal of publication, study design, and patient characteristics, including age, gender, weight, height, and BMI. The post-operative day (POD) when symptoms of SMAS started was recorded. Data regarding the type of surgical scoliosis correction, signs and symptoms, diagnosis and management of SMAS were retrieved. Data were extracted in Microsoft Office Excel version 16.72 (Microsoft Corporation, Redmond, USA).

### Assessment of the risk of bias

The risk of bias was examined following the guidelines in the Cochrane Handbook for Systematic Reviews of Interventions [25]. The Nonrandomised Studies of Interventions (ROBINS-I) tool was used for all included non-RCTs [26]. Seven domains of potential bias in non-RCTs were assessed. Possible confounding and the nature of patient selection before the comparative intervention are evaluated by two domains. A further domain is used to evaluate bias in the classification during the intervention. The final four domains assess the methodological quality after the intervention comparison has been implemented and relate to deviations from previously intended interventions, missing data, erroneous measurement of outcomes, and bias in the selection of reported outcomes. The figure of the ROBINS-I was elaborated using the Robvis Software (Risk-of-bias VISualization, Riskofbias.info, Bristol, UK) [27]. Case reports included in this investigation were evaluated using the Joanna Briggs Institute (JBI) criteria appraisal tools for case reports [28]. Possible answers were “yes”, “no”, “unclear”, or “not applicable”. Eight questions were answered to evaluate the methodological quality in accordance with the JBI checklist. The included studies were assessed based on the following bias: low risk of bias: studies with more than six “yes”; moderate risk of bias: studies with five to six “yes”; and high risk of bias: studies with less than five “yes”. Two reviewers (G.P. and L.S.) evaluated the risk of bias in the extracted studies separately. Disagreements were solved by a third senior author (F.M.).

## Results

### Study selection

**Fig. 1:**
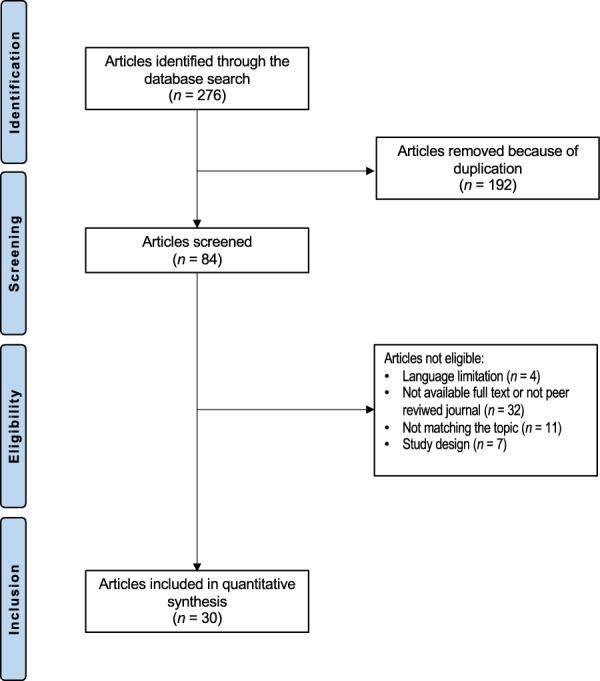
PRISMA flow chart of the literature search

The literature search resulted in 276 articles. Of them, 192 were excluded as they were duplicates. Additionally, 44 studies were excluded for the following reasons: study design (*N* = 7), low level of evidence (*N* = 6), full text not available or not in peer-reviewed journals (*N* = 16), and language limitations (*N* = 4). An additional 11 studies were excluded because they did not offer quantitative data on the outcomes of interest. Finally, 29 articles were included in the present investigation. Of them, nine had a retrospective study design, and 20 were case reports. The results of the literature search are visible in Fig. 1.

### Methodological quality assessment

**Fig. 2:**
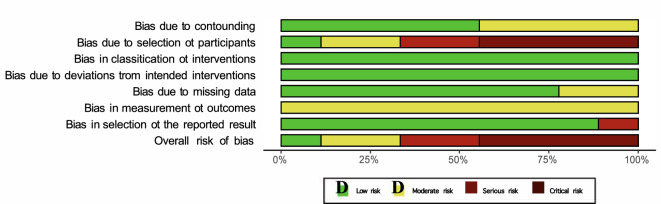
ROBINS-I of non-RCTs

The ROBINS-I was applied to investigate the risk of bias in 31.0% (9 of 29) of the included studies, as they were non-RCTs. Given the small number of patients studied in the included investigations, the risk of bias based on confounding and the selection of participants was rated in four studies critical because they reported data from only one patient. The residuary studies were rated with a moderate to serious risk of bias in the cited domains. The intervention protocol was well reported in most studies, and no significant deviation from the interventions was identified, showing a primarily low risk of bias in the classification of interventions and deviation from intended interventions. Data were adequately reported in the most included studies, and the measurement of the outcomes was equivalent among the groups. Given the lack of randomisation of the investigators and patients, the measurement of the outcomes was evaluated with a moderate risk of bias in all of the studies. The reported results corresponded to the planned protocol in most included studies. Given the overall poor methodological quality in the included studies, the overall risk of bias was predominantly serious to critical. The assessments of the methodological quality are given in Fig. 2.

**Fig. 3:**
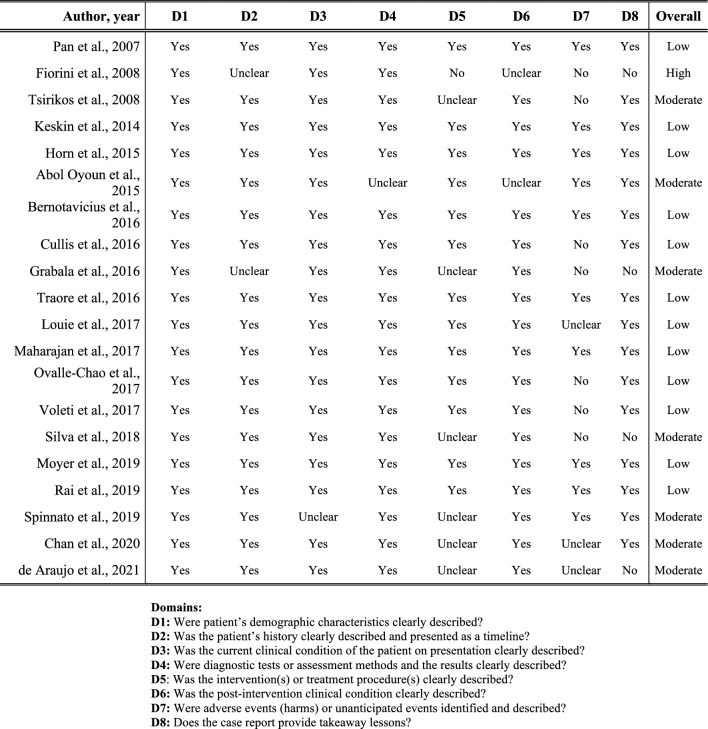
JBI criteria appraisal tool for case reports

The risk of bias using the JBI criteria appraisal tool for case reports was evaluated in 69.0% (20 of 29) of the included studies, as they were case reports. While seven of the included studies received a score of six and five, respectively, indicating moderate quality, 12 received a score of seven or eight, demonstrating good-quality evidence. The case report by Fiorini et al. received a score of three out of eight, suggesting low-quality evidence with a high risk of bias. The detailed assessment steps are shown in Fig. 3.

### Study characteristics and results of individual studies

A total of 61 patients were included in the present study. 62% (38 of 61 patients) were women. The mean age was 15.8 ± 7.2 years. The mean weight was 45.3 ± 8.0 kg, the mean height 159.6 ± 13.6 cm, and the mean BMI 16.5 ± 2.9 kg/m^2^. The generalities and demographics of the included studies are shown in Table 1.

**Table 1:** Patient generalities of the included studies

Authors, year	Journal	Study design	Patients (n)	Mean age (y)	Mean BMI (*kg/m*^*2*^)	Mean POD
Crowther et al. [19]	*Spine (Phila)*	Retrospective	3	15		6
Shah et al. [17]	*J Ped Orth*	Retrospective	6	15		50
Altiok et al. [18]	*Spine (Phila)*	Retrospective	17	15	19	7
Tsirikos et al. [29]	*J Spinal Disor*	Retrospective	2	14		6
Zhu et al. [30]	*World Journal Gastr*	Retrospective	1	15		5
Hod-Feins et al. [11]	*J Pediatr Orth*	Retrospective	7	14		6
Pan et al. [31]	*J Formos Med Assoc*	Case report	2	12	13	3
Fiorini et al. [32]	*Arch Argent Pediatr*	Case report	1	18		366
Tsirikos et al. [8]	*J Med Case Rep*	Case report	1	17	21	45
Smith et al. [33]	*J Spinal Disor Tech*	Retrospective	1	15	19	18
Keskin et al. [34]	*Case Rep Surg*	Case report	1	17		5
Horn et al. [35]	*An J Orthop*	Case report	1			14
Abol Oyoun et al. [36]	*SICOT J*	Case report	1	12	15	1464
Bernotavicius et al. [37]	*Acta Med Lit*	Case report	1	12	17	4
Cullis et al. [38]	*Scott Med J*	Case report	1	12		
Grabala et al. [39]	*Pol Ann Med*	Case report	1	13	19	5
Traore et al. [40]	*Pan Afr Med J*	Case report	1	25	14	4
Eisenson et al. [41]	*R I Med J*	Retrospective	1	15	12	14
Louie et al. [1]	*Am J Orthop*	Case report	1	14	15	19
Maharajan et al. [42]	*J Spine Surg*	Case report	1	13	16	35
Ovalle-Chao et al. [43]	*J Ped Surg Case Rep*	Case report	1	14	13	1
Voleti et al. [44]	*J Orthop Case Rep*	Case report	1	17	20	14
Silva et al. [45]	*Rev Esp Enferm Dig*	Case report	1	15		4
Moyer et al. [46]	*JBJS Case Conn*	Case report	1	11		6
Rai et al. [2]	*J Bone Joint*	Case report	1	13	18	6
Spinnato et al. [47]	*Ped int*	Case report	1	13		2
Chan et al. [48]	*J orth Surg*	Case report	1	16		7
Chung et al. [20]	*J Orth Surg*	Retrospective	2	16	16	10
de Araujo et al. [49]	*Rev Bras Ortop*	Case report	1	12	16	11

### Synthesis of results

The mean interval between spine surgery and symptoms occurrence was 69.1 ± 262.4 days (3 days to 4 years). The mean duration of the treatment for SMAS was 21.6 ± 10.3 days. Table 2 provides an overview of the main findings of the included studies.

**Table 2:** Overview of the main findings of the included studies

Year, Author	Surgical managment	Sings and symptoms	Diagnostic	Treatment	Conclusion
Crowther et al. [19]	ASIF, PSIF	Nausea, bilious vomiting, abdominal distension	Upper GI: straight-line obstruction at the duodenum (D3)	Nasogastric decompression; nasojejuneal feeding, parenteral nutrition, laparotomy	Vomiting following correction scoliosis surgery should be investigated rapidly and meticulous, as SMAS causes relevant morbidity, and potentially death
Shah et al. [17]	PSIF	Incessant vomiting	Upper GI: straight-line obstruction at the duodenum (D3)	Laparotomy and duodenojejunostomy, nasojejunal feedings, prone positioning	The 5th percentile defines the degree of asthenia necessary for critical compromise of the arteriomesenteric angle: this group of patients may be predisposed to vascular compression of the duodenum
Altiok et al. [18]	ASF, PSIF	Nausea, vomiting	Abdominal XR via barium swallow or injection of contrast via a nasogastric tube: abrupt vertical cutoff at the third portion of the duodenum by the SMA and aorta, around the duodenum	Nasogastric decompression nasojejunal feeding parenteral hyperalimentation	Low BMI and perioperative weight loss represent to be the two most important risk factors for identifying patients at risk of SMAS
Tsirikos et al. [29]	ASF, PSIF	Nausea, vomiting, abdominal pain and distension	Abdominal US: distension of the stomach, dilatation D1 and D2	Nasojejunal tube	Conservative treatments should be used as initial treatment reducing duodenal compression by the SMA and reducing the intestinal edema
Zhu et al. [30]	ASF, PSIF	Nausea, bilious vomiting, abdominal pain and distension, loss of appetite, hypoactive bowel sounds	Upper GI: straight-line obstruction at the duodenum (D3)	Fasting, antiemetic medication, intravenous fluid infusions	Height percentile < 50%, weight percentile < 25%, sagittal kyphosis, heavy and quick halo-femoral traction after spinal anterior release are the potential risk factors for SMAS
Hod-Feins et al. [11]	ASF, PSIF	Nausea, bilious vomiting, abdominal pain, constipation	Upper GI: proximal duodenal dilation and obstruction at D3 Abdominal XR: gas filling the rectum and gas–liquid levels	Nasogastric decompression, total parenteral nutrition	Reduction of the lumbar lordosis after anterior fusion, leads to a widening of the aortomesenteric angle, which should have been a protective factor
Pan et al. [31]	ASIF, PSIF	Abdominal distension and pain, hypoactive bowel sounds	Abdominal XR: left distended bowel gas; Abdominal US: negative increase serum amylase and lipase	Nasogastric decompression	Even though SMAS represents a rare complication after scoliosis’ correction in AIS, deferred or not appropriable diagnosis could lead to fatal consequences
Fiorini et al. [32]	PSIF	Bilious vomit, abdominal pain, weight loss	Upper GI: obstruction at the duodenum (D3) Abdominal sonography: microlitiasis	Hypercaloric diet lateral decubitus	Upper gastrointestinal barium-contrast radiography could be used for diagnosis of SMAS. Surgery is needed when conservative measures are not effective
Tsirikos et al. [8]	ASIF	Nausea, vomiting, abdominal distension, oliguria, dehydration, electrolyte abnormalities	Upper GI: dilatation of the stomach and proximal duodenum, occlusion third part of the duodenum (D3)	Nasogastric decompression nasojejunal tube	Progressive postoperative weight loss seems to be a critical factor in the presentation and occurrence of SMAS
Smith et al. [33]	PSIF	Nausea, vomiting, anorexia, weight loss	Upper GI: obstruction at the level of the duodenum	Nasojejunal feeding	Patients with preoperative BMI < kg/m^2^ have a major risk to develop SMAS postoperatively
Keskin et al. [34]	PSIF	Nausea, vomiting, abdominal distension, weight loss	Upper GI: obstruction between D2 and D3 Abdominal XR: air fluid level Abdomen contrast CT: dilatation of the stomach, D1 and D2	Nasogastric decompression, laparotomy: side to side duodenojejunostomy	Following scoliosis surgery, SMAS SMAS is provoked by the consequent reduction of the aortomesenteric angle and compression of the SMA trunk
Horn et al. [35]	PSIF	Bilious vomiting, abdominal pain, increase of serum lipase	Abdominal XR: stomach's distension and loop of small bowel below the liver with and air fluid level Upper GI: air fluid level in the stomach, dilatation of the second portion of the duodenum, reflux of contrast material into the stomach from duodenum with no passage of barium into the distal duodenum	Nasojejunal feeding gastrojejunostomy	Physicians should be alert for signs and symptoms of SMAS in patients with preoperative low BMI and increased thoracic stiffness
Abol Oyoun et al. [36]	PSIF	Persistent abdominal pain, weight loss	Upper GI: reduction of the contrast motility between the third and second portions of the duodenum	Hypercaloric diet	Following scoliosis correction surgery for AIS, even with non-GI-related symptoms, SMAS should be evaluated as possible diagnosis
Bernotavicius et al. [37]	PSIF	Nausea, vomiting, electolyte abnormalities	Clinical diagnosis	Nasogastric decompression, electrolytes correction with intravenous fluids	In patients with preoperatively low BMI, the identification of factors of the SMAS and begin preoperative diet supplements before surgery should be recommended
Cullis et al. [38]	PSIF	Vomiting, weight loss, abdominal distension	Upper GI: incomplete duodenal obstruction Abdominal CT: reduced aorto-mesenteric distance Gastroscopy:mild gastritis	Laparoscopic duodeno-jejunostomy	A high success surgical treatment of SMAS is represented by laparoscopic duodeno-jejunostomy
Grabala et al. [39]	PSIF	Nausea, vomiting, abdominal pain and distension	Contrast CT abdomen: the angle of the SMA ramification from the aorta is approximately 17°, the level of the duodenum is of 4.3 mm to 6.5 mm between the SMA and the aorta reduction of the angle of the SMA ramification from the aorta	Parenteral nutrition	Preoperative BMI < 19 kg/m^2^ with postoperative elongated axis of the spine, seems to be important risk factors for SMAS
Traore et al. [40]	PSIF	Vomiting, abdominal pain and distension, electrolyte abnormalities	Abdomen contrast CT: obstruction at D3	Nasogastric decompression parenteral nutrition	If SMAS is rapidly and consequently diagnosticated and treated, the prognosis is positive
Eisenson et al. [41]	PSIF	Bilious vomiting, weight loss	Upper GI: hold up contract at D3 with dilatation of proximal duodenum and stomach	Nasojejunal tube, nasogastric decompression, nasogastric feeds, total parenteral nutrition, per os diet	Bilious vomiting and abdominal pain are the most common clinical symptoms of SMAS after correction spinal surgery in AIS patients
Louie et al. [1]	PSIF	Nausea, braun vomiting, abdominal pain, weight loss	Upper GI: contrast held up at D3 Abdominal US: nonspecific fluid-filled bowel loops	Nasojejunal tube	Early recognition of the nonspecific abdominal symptoms, bilious vomiting, hypoactive bowel sounds could lead to timely diagnosis of SMAS
Maharajan et al. [42]	PSIF	Vomiting, abdominal pain, dehydration, weight loss	Upper GI: dilatation and stasis of contrast in the third segment of duodenum (D3)	Nasojejunal feeding antiemetics intravenous fluids	By timely diagnosed SMAS, the conservative management shows good clinical outcomes
Ovalle-Chao et al. [43]	PSIF	Nausea, bilious vomiting, abdominal pain	Upper GI: D3 obstruction with slight dilatation of D2 CTA: narrowed aortomesenteric angle Upper endoscopy: complete obstruction of duodenum obstruction with slight dilatation of D2	Nasogastric decompression, duodenum–jejunum bypass	Low BMI and low percentile of weight for height are risk factors for SMAS in AIS patients after scoliosis surgery. Continuously bilious vomiting should increase the suspection of intestinal occlusion
Voleti et al. [44]	PSIF	Nausea, vomiting, abdominal pain and distension, dehydration, electrolyte disorders, metabolic alkalosis, weight loss	Upper GI and abdominal ultrasound positive for SMAS Abdominal CTA: narrowed aortomesenteric angle and narrowed aortomesenteric distance	Nasogastric decompression, total parenteral nutrition, correction of dyselectrolytemia, gastrojejunostomy	Postoperative upper gastrointestinal symptoms along with weight loss, represents to have a clinical key-role in the diagnosis of SMAS
Silva et al. [45]	PSIF	Nausea, vomiting, anorexia	Abdominal XR: distended stomach with intra-abdominal air Abdominal CT: narrowed aortomesenteric angle and narrowed aortomesenteric distance duodenal obstruction with abrupt cutoff in D3 Upper GI: extrinsic compression of D3	Nasojejunal feeding	If SMAS is rapidly and consequently diagnosticated and treated, the prognosis is positive
Moyer et al. [46]	PSIF	Vomiting, abdominal pain and distension	Abdominal XR: distended stomach Abdominal CT: massively distended stomach and distention of D1 and D2, extensive gastric and duodenal pneumatosis Endoscopy (POD6): partial-thickness necrosis of the stomach mucosa Endoscopy (POD11): resolution of the partial necrosis but continued marked gastric distension	Nasogastric decompression total parenteral nutrition nasogastric feeds postopyloric feeding tube	Underlying cardiovascular, neurologic, or gastrointestinal findings may contribute to the severity of SMAS
Rai et al. [2]	PSIF, Anterior transthoracic release	Nausea, vomiting, abdominal pain	Abdominal CT: obstruction of D3 in the aortomesenteric angle	Nasogastric decompression nasojejunal feeding gastric decompression parenteral nutrition antiemetic laparoscopic duodenojejunostomy	If timely diagnosed, SMAS should be managed with conservative medical treatment. Surgery reserved for nonresponders
Spinnato et al. [47]	PSIF	Vomiting, abdominal pain	Abdominal XR: severe gastric distension with a huge air–liquid level; The D1 was distended while other small bowel loops were difficult to visualize Abdominal CTA: compression of D3 between the SMA	Nasogastric decompression parenteral nutrition enteral nutrition	Reported clinical presentation and radiological findings, in association with predisposing factors, should be considered pathognomonic for SMAS
Chan et al. [48]	PSIF	Vomiting, abdominal pain	Abdomen CT: dilated stomach and first and second part of duodenum Abdominal CTA: narrowed aortomesenteric angle and narrowed aortomesenteric distance	Nasogastric tube correction of electrolytes nutritional support	If approrpiarly diagnosed, SMAS responds well to the conservative medical treatment
Chung et al. [20]	PSIF	Vomiting, abdominal distension	Abdominal XR: distended gastric shadow and fluid level Abdominal CTA: narrowed aortomesenteric angle and narrowed aortomesenteric distance	Nutritional support electrolytes imbalance correction	Patients with SMAS can be treated successfully with conservative treatment including nasogastric decompression, electrolyte correction, and nutritional support with small but frequent meals
de Araujo et al. [49]	PSIF	Vomit, abdominal distension, loss of appetite	Upper digestive endoscopy	Laparotomy and duodenal shunt nasogastric tube	Postoperative elongated axis of the spine changes the anatomical surroundings of the SMA, which seems like to be a potential risk factors for the syndrome

*ASIF* anterior spinal instrumentation and fusion, *PSIF* posterior spinal instrumentation and fusion, *CT* computer tomography, *CTA* computed tomography angiography, *D1* first (transversal) part of the duodenum, *D2* descend part of the duodenum, *D3* third (horizontal) part of the duodenum, *SMAS* Superior mesenteric artery syndrome, *SMA* superior mesenteric artery, *MIS* minimally invasive surgery, *POD* postoperative day, *XR* X-ray, *Upper GI* upper gastrointestinal barium contrast study with concomitant radiography, *US* ultrasound, *PSF* posterior spinal fusion

## Discussion

The systematic review found that the mean interval between spine surgery and symptoms occurrence was 69 days, with high between-studies variability (3 days to 4 years). The mean duration of the management for a diagnosed SMAS was 21.6 days.

Following spinal surgery in AIS, SMAS is a complication provoked by extrinsic compression of the third part of the duodenum as it crosses between the SMA, which lies anteriorly, and the abdominal aorta and lumbar spine posteriorly [7, 13, 50–52]. The SMA supplies blood to the entire small intestine except the duodenal bulb, cecum, ascending, and transverse colon. The vessel arises from the anterior aspect of the aorta at the level of the L1 vertebral body and extends caudally into the small bowel mesentery [53]. The third part of the duodenum passes between the aorta and SMA and is suspended by the ligament of Treitz, also known as the suspensory muscle of the duodenum [54–57]. The aortomesenteric angle and the aortomesenteric distance usually range from 28° to 65° and 10 to 34 mm, respectively. In SMAS, they are reduced, having values of 6–15° and 2–8 mm, respectively [13, 51, 58–61]. The third part of the duodenum is generally surrounded by periduodenal fat at the aortomesenteric angle. Modifying this anatomy could lead to vascular compression of the third part of the duodenum, which might lead to SMAS [7, 8, 13, 41, 62]. The risk factors for SMAS can be congenital or acquired, and identifying patients at risk for SMAS is pivotal to avoiding complications. A congenital short ligament of Treitz could pull the duodenum upward into the vascular angle between the SMA and the aorta, causing vascular compression [11, 30]. Acquired causes related to a decrease in intestinal padding include weight loss (weight < 25% percentile, BMI < 25th percentile), eating disorders, cachexia, severely debilitating conditions, and incapacitating illnesses such as malignancies [22, 63–65]. A lower BMI, height, or weight than 5%, 50%, or 25% percentile, sagittal kyphosis, and heavy and quick halofemoral traction after anterior spinal release are other important risk factors for SMAS [41]. Acquired and iatrogenic modifications of the aortomesenteric angle due to rapid height gain (> 50% percentile) or postoperative spinal lengthening could also lead to SMAS. Children mostly attend a surgical correction of AIS in their most rapid longitudinal growth phase. Moreover, children or adolescents with scoliosis and thoracic curves also have hyperkyphosis, which leads to a more extended spine and reduces the aortomesenteric angle [8, 11, 18, 19, 29, 30, 41]. Willner et al. [66] showed that a spurt of growth is particularly accelerated in the year before the diagnosis of AIS, and these patients tend to be taller and more slender than their age-matched counterparts. Moreover, there may be an acute increase in vertebral column length after posterior and anterior spinal instrumentation [67, 68]. The traction on the SMA and, consequently, narrowing of the aortomesenteric angle can be caused by the acute increase in vertebral column length following spinal instrumentation. Finally, postoperative weight loss causes retroperitoneal fat reduction that protects the duodenum from compression [8, 11, 18, 19, 29, 30, 41]. During the retroperitoneal dissection in the anterior approach to the thoracolumbar spine, the disruption of the autonomic nerve supply to the small intestine may also trigger the development of the condition [19]. The mean interval between spine surgery and the symptoms of SMAS was 69 days, with high variability between studies (3 days to 4 years). Prompt identification of risk factors and an early diagnosis are necessary to manage SMAS and reduce the risk of complications. Delay in the development of the syndrome might be related to the progressive weight loss occurring in the postoperative period, resulting in gradual loss of the retroperitoneal fat [42, 69, 70]. Symptoms include nausea, anorexia, bilious vomiting, and intestinal constipation, with partial relief of them with postural changes and epigastric pain [71–74]. During the physical examination, the peristalsis can be normal or hyperkinetic, and the abdomen is soft with occasional tenderness in the epigastrium at deep palpation [19, 41]. Symptoms of SMAS are similar to paralytic ileus [18, 41, 68, 75]. Recurrent vomiting with gastric dilatation could eventually lead to severe hypovolemia, progressive dehydration, oliguria, and electrolyte disorders, such as hypokalaemia and metabolic alkalosis, or even death [68, 76, 77]. Death in SMAS can result from chemical pneumonia by vomitus inhalation or gastric perforation [8, 44, 78].

SMAS is characterised by three criteria: dilated duodenum, reduction of the aortomesenteric angle to less than 25°, and compression of the third part of the duodenum by the SMA [13, 77, 79–81]. Barium imaging can assist in the diagnosis, showing the characteristic duodenal dilatation with an abrupt vertical cutoff in the third part of the duodenum and a 4–6-h delay in the gastroduodenal transit [22, 82]. The gold standard radiological methods in SMAS diagnosis is represented by computer tomography angiography (CTA), which reveals compression of the third part of the duodenum by the SMA with subsequent proximal duodenal dilatation and a reduced aortomesenteric angle and aortomesenteric distance [61, 62, 83, 84]. In most patients, conservative management is associated with improving the symptoms, usually occurring after 2–3 days [85]. Oral intake should be restricted, and under radiographic assistance, nasojejunal feeding should be passed distally from the duodenal obstruction to achieve enteral feeding and progressive weight gain with high-caloric nutritional supplements [85]. If the symptomatology persists or enteral feeding is impossible, total parenteral nutrition should be started.

Failure of the conservative treatment can lead to life-threatening conditions such as metabolic alkalosis, electrolyte imbalance, and aspiration pneumonia [13, 20, 86, 87].

If conservative management fails, gastrojejunostomy, duodenojejunostomy, or Ladd procedure is advocated [88–91]. Although conservative management is the first therapeutic approach in patients with SMAS, the current evidence lacks large clinical trials and recommendations. An internationally accepted therapeutic algorithm for the management of SMAS is necessary. Given the rarity of SMAS, the current evidence is mostly case reports. Therefore, the generalisation of these results is limited, and an estimate of the prevalence of SMAS is unreliable. Moreover, the limited evidence hinders the establishment of reliable risk factors and a proper algorithm for diagnosing and treating SMAS. Additional investigations are required to establish risk factors and diagnostic criteria.

## Conclusion

SMAS is a complication of spinal surgery in AIS caused by extrinsic compression of the third part of the duodenum. The mean interval between spine surgery and symptoms occurrence was 69 days, with high between-studies variability (3 days to 4 years). Prompt identification of risk factors and an early diagnosis are necessary to manage SMAS and reduce the risk of complications. Additional investigations are required to establish risk factors and diagnostic criteria.

### Supplementary Information

Additional file 1.

## Data Availability

The datasets generated during and analysed during the current study are available throughout the manuscript. No datasets were generated or analysed during the current study.

## Abbreviations

ASIF: Anterior spinal instrumentation and fusion
PSIF: Posterior spinal instrumentation and fusion
CT: Computer tomography
CTA: Computed tomography angiography
D1: First (transversal) part of the duodenum
D2: Descend part of the duodenum
D3: Third (horizontal) part of the duodenum
SMAS: Superior mesenteric artery syndrome
SMA: Superior mesenteric artery
MIS: Minimally invasive surgery
POD: Postoperative day
