# Supplementary material for: Superior mesenteric artery syndrome following spine surgery in idiopathic adolescent scoliosis: a systematic review
Source: Eur J Med Res. 2024 Aug 8;29:410. doi: 10.1186/s40001-024-02002-3 (PMC11308422; doi:10.1186/s40001-024-02002-3)
Supplement: Supplementary file 1 — Additional file 1. [file 40001_2024_2002_MOESM1_ESM.docx]

**Systematic literature search for:**

**Superior mesenteric artery syndrome following spine surgery in idiopathic adolescent scoliosis: a systematic review**

**Concept 1:**

**Keywords:**

Spine

scoliosis

spondylodesis

**Mesh:**

"Spine"[Mesh]

"Spine/pathology"[Mesh]

"Spine/surgery"[Mesh]

"Scoliosis"[Mesh]

"Spinal Fusion"[Mesh]

**Concept 2:**

**Keywords:**

superior mesenteric artery syndrome

SMAS

Wilkie syndrome

Cast syndrome

mesenteric duodenal compression syndrome

**Mesh:**

"Superior Mesenteric Artery Syndrome"[Mesh]

**Concept 1:**

"Spine"[Mesh] OR "Spine/pathology"[Mesh] OR "Spine/surgery"[Mesh] OR "Scoliosis"[Mesh] OR "Spinal Fusion"[Mesh] OR Spine OR scoliosis OR spondylodesis

**AND**

**Concept 2:**

"Superior Mesenteric Artery Syndrome"[Mesh] OR superior mesenteric artery syndrome OR SMAS OR Wilkie syndrome OR Cast syndrome OR mesenteric duodenal compression syndrome

**Summary of the Search**

("Spine"[Mesh] OR "Spine/pathology"[Mesh] OR "Spine/surgery"[Mesh] OR "Scoliosis"[Mesh] OR "Spinal Fusion"[Mesh] OR Spine OR scoliosis OR spondylodesis) AND ("Superior Mesenteric Artery Syndrome"[Mesh] OR superior mesenteric artery syndrome OR SMAS OR Wilkie syndrome OR Cast syndrome OR mesenteric duodenal compression syndrome)
